# Alleviative effects of Fara-darmani Consciousness Field on *Triticum aestivum *L. under salinity stress

**DOI:** 10.12688/f1000research.25247.2

**Published:** 2020-12-02

**Authors:** Sara Torabi, Mohammad Ali Taheri, Farid Semsarha

**Affiliations:** 1 Department of Plant Biology, School of Biology, College of sciences, University of Tehran, Tehran, 14155-6455, Iran; 2 Sciencefact Research Center, , Cosmointel Ltd. Research Center, Ontario, Canada, Ontario, Canada; 3 Institute of Biochemistry and Biophysics (IBB), University of Tehran, Tehran, 13145-1384, Iran

**Keywords:** Antioxidant enzymes, Fara-darmani, Consciousness Field, Salt stress, Wheat

## Abstract

**Background**: The Fara-darmani Consciousness Field was founded by Mohammad Ali Taheri. It is a novel field and is described similarly to the field of gravity, or the electromagnetic field. This field is neither matter nor energy, and therefore does not possess a quantity. Even though there is no direct scientific evidence for the Consciousness Field, it is possible to investigate its effects on objects through controlled experiments. The aim of the present work was to study the alleviative effects of the Fara-darmani Consciousness Field on common wheat *Triticum aestivum L*. var Star under salt stress.

**Methods**: Plants were grown under 0 mM NaCl (control) and 150 mM NaCl with or without the influence of Fara-darmani Consciousness Field for 3 weeks. Chlorophyll, hydrogen peroxide (H _2_O _2_), malondialdehyde (MDA) content and activity of antioxidant enzymes such as superoxide dismutase (SOD),polyphenol oxidase (PPO), and peroxidase (POX) were measured in all groups of plants.

**Results**: In the salt-treated plants under the influence of the Fara-darmani Consciousness Field, the contents of total chlorophyll, as well as a and b chlorophyll forms, were elevated compared with the salt-treated plants without Fara-darmani CF (34.8%, 17.8%, and 169% respectively). Additionally, Fara-darmani increased H _2_O _2 _(57%) and the activity of SOD and PPO by 220% and 168%, respectively, under salinity compared with the salt-treated plants without Fara-darmani CF. MDA content and activity of peroxidase were decreased by 12.5% and 34%, respectively.

**Conclusion**: These results suggest the Fara-darmani Consciousness Field as a qualitative intervention strategy to withstand salt stress in plants, by increasing the contents of chlorophyll, antioxidant enzyme activities, and decreasing MDA content under salinity.

## Introduction

Most criticism about complementary therapy is the lack of scientific research. In order to be accepted, academic studies using different study designs are necessary. Since one of the critical objections occurring when human beings are treated with complementary therapy is the placebo responses, biochemical plant-based studies can be a suitable method to clarify the phenomenon ( Betti *et al*., 2003). Among the different plant model systems, the wheat plant has been repeatedly selected for homeopathy research Baumgartner *et al*. (2000) showed that homeopathic drugs improved plant resistance, which exerted their effect through detoxification processes.

In arid and semi-arid areas of the world, salinity is considered as a major factor in reducing crop productivity ( Poonia *et al*., 1972). Plant growth is adversely affected by multiple environmental stresses, including biotic (e.g. fungi, bacteria, viruses, herbivores) and abiotic (e.g. low temperature, salt, drought, heavy metal toxicity). Among these the salination of arable land is one of the key factors that threatens the sustainability of the agricultural industry. Thus, many studies have attempted to explore processes that contribute to plant survival under salt stress ( Ashraf & Harris, 2004) as a strategy to improve productivity and fertility. It is well documented that plants that are exposed to biotic or abiotic stresses have biochemical changes that exert oxidative damage through Reactive Oxygen Species (ROS) ( Smirnoff, 1993). These free radicals disrupt cell membrane stability by peroxidation of polyunsaturated fatty acids in the plant cell membranes ( Bor *et al*., 2003; Hernández & Almansa 2002; Shalata *et al*., 2001) and denature protein and nucleic acids ( Chen *et al*., 1993). To alleviate adverse effects of oxidative stress, plants have developed diverse strategies, which are categorized as enzymatic, such as catalase, superoxide dismutase (SOD), peroxidase (POX), polyphenol oxidase (PPO) and ascorbate peroxidase, and non-enzymatic that directly scavenge ROS, such as glutathione, tocopherol, flavonoids and ascorbates ( Agarwal & Pandey, 2004). Plants that have developed an antioxidant system that participates in ROS scavenging have better resistance to oxidative damage ( Parida & Das, 2005).

To date, there have been many studies to explore the relationship between the intangible and physical world, especially the interaction between the human mind and outside physical world. For instance, it has been reported that the mind can affect dice tosses ( Rhine, 1944). Researchers have previously focused on probabilistic systems, like tossing coins, using random number generators (RNGs). The first RNG study was conducted by Radin & Nelson (1989), which included 597 experiments and 235 control studies. This type of research was considered as ‘micro-psychokinesis’ (micro-PK) ( Jahn *et al*., 1980; Varvoglis & Bancel, 2015). However, micro-PK is not completely acceptable to science because of the null effects and failure to replicate previous positive results ( Jahn *et al*., 2000). Throughout history, studies can be found that explain the interaction between the human mind and body, such as ‘distant healing’, or the effects of the mind on inanimate physical systems, like morphological changes in a thin strip of metal ( Randall & Davis, 1982).

Fara-darmani is one of the many Consciousness Field (CFs) founded by Mohammad Ali Taheri. In this theoretical concept, cosmic consciousness is the collection of consciousness, wisdom or intelligence governing the world of existence, which is also called ‘Awareness’. Consciousness, according to Taheri, is one of the three existing elements of the universe apart from matter and energy. By defining Consciousness as neither matter nor energy, we cannot associate a quantity to it. Since consciousness isn’t measurable, its existence can only be known through experience ( Taheri, 2013). According to this theory, any living creature, including animals and plants, may be cured via humans by connecting to internet-like facilities called the Cosmic Consciousness Network (CCN). In this type of affection, mind-matter interaction occurs through connecting to the CCN by a Fara-therapist. Fara-darmani establishes a consciousness bond between the ‘whole’ consciousness and the ‘parts’ where all constituents will be scanned and corrected ( Taheri, 2013). Although the mechanism of this linkage is not yet definable by science, its consequences can be measured and studied scientifically.

The aim of this study was to determine the effects of Fara-darmani CF on alleviating the effects of salt stress in a spring wheat variety (Star).

## Methods

### Fara-darmani Consciousness Field application

In Fara-darmani, subjects of study become connected to that Consciousness Field via Fara-therapist by ‘announcement’ which is a process in which Taheri or any Fara-therapist (announcer) declares and sends the information of the subjects under study (e.g. the number of groups) to the CCN. The influence of Consciousness Field begins with the connection between the human mind and the CCN. In other words, the Fara-therapist’s mind acts as an intermediary between the subject of the study and the CCN. The first author of this study is an announcer and at the same time as the seedlings were subjected to salt stress, two groups of treatments became connected to the CCN (group 2: 0 mM NaCl and group 4: 150 mM NaCl). This exposure occurs without any kind of physical intervention, since consciousness according to Taheri’s concepts is neither matter nor energy, receiving this treatment is possible from close and far distances.

This experiment can easily be repeated by any researcher even from far distances by registering on the COSMOintel website (the Assign Announcement section) COSMOintel is a research center, under the supervision of the innovator of the method (Mohammad Ali Taheri) that has been established to design and implement repeatable and reproducible studies in the world of science ^1^.

### Plants

In this research, we used a spring wheat variety *Triticum aestivum L*. var Star (Seed and Plants Improvement Institute, Karaj, Alborz Province). Seeds were surface sterilized with 2.5% sodium hypochlorite for 10 min and washed thoroughly with sterile distilled water. After sterilization, seeds were soaked in distilled water for 24 hours at room temperature. For each treatment three pots were prepared and six seeds were initially sown in plastic pots (10 × 10 cm) containing perlite soil. After the germination they were thinned to five plants per pot. First, all pots were irrigated daily with 100ml distilled water for four days. Then received 100ml half-strength Hoagland’s nutrient solution (pH= 5.7) ( Hoagland & Arnon, 1950) every other day for another 12 days (chemicals purchased from Sigma-Aldrich).

### Applying salinity stress

 The sixteen-day-old seedlings were treated with salinity. The salts were added to the nutrient solution. To prevent osmotic shock, salt stress was started gradually on 50 mM NaCl (100ml). Every other day the concentration was increased by 50mM until 150 mM was attained. Salt stress was continued for three weeks (150mM NaCl was added every other day). Initial Fara-darmani connection treatment occurred at the same time as adding the first NaCl solution. Four treatment groups (n=3 pots/group) were performed as follows: group 1, control – grown with no NaCl and did not receive Fara-darmani CF; group 2 –grown with no NaCl and did receive Fara-darmani CF; group 3 – treated with 150 mM NaCl for three weeks and did not receive Fara-darmani CF treatment; group 4 – treated with 150 mM NaCl for three weeks and did receive Fara-darmani CF treatment.

After three weeks, four fully expanded leaves were picked per replicate for future analyses. They were frozen in liquid N _2_ and transferred to -20˚C for imminent bench experiments.

### Determination of chlorophyll content

For measuring photosynthetic pigments, we used the method by Arnon (1949). 0.5 gram of fresh leaf material placed in acetone 80% and homogenized to extract chlorophyll. The resulting solution was filtered through Whatman’s No.1 filter paper. After extracting of photosynthetic pigments in acetone 80%, absorbance of chlorophyll a and b was recorded by UV-visible spectrophotometer (Shimadzu UV-160) at 645 and 663 nm respectively. According to Arnon (1949) chlorophyll concentrations were calculated using the formulas below:

Chl.a (mg l ^-1^) = [12.7 (A _663_) – 2.69 (A _645_)] * 0.5 ml of extracted sample

Chl.b (mg l ^-1^) = [22.9 (A _645_) – 4.69 (A _663_)] * 0.5 ml of extracted sample

Total chlorophyll = Chl a + Chl b

### Determination of hydrogen peroxide content

Measurement of the hydrogen peroxide (H _2_O _2_) content was performed according to Velikova *et al*. (2000). One gram of leaf tissue was homogenized on ice with 5 ml of trichloroacetic acid (TCA; Sigma-Aldrich) 0.1% (w/v) and centrifuged at 12000 rpm for 15 min. Subsequently, 0.5 ml of 10 mM potassium phosphate buffer (pH 7) and 1 ml of 1M potassium iodide was added to 0.5 ml of supernatant. The absorbance of supernatant was determined at 390 nm wavelength.

### Determination of lipid peroxidation

Malondialdehyde (MDA), which is a product of lipid peroxidation, has been considered as an indicator of membrane destruction. MDA content was determined according to Stewart & Bewley (1980). We added 5 ml of TCA to 0.2 g of fresh leaf. After homogenization, the solution was centrifuged at 13000 × g for 10 min. The mixture of 1 ml of supernatant with 4 ml of 0.5% thiobarbituric acid in 20% TCA was heated for 30 min at 95˚C and quickly placed in an ice bucket. Subsequently, we centrifuged the solution at 10000 × g for 10 minutes and recorded the absorbance of supernatant at 532 and 600 nm. The calculation of MDA was done from the extinction coefficient of 155 mM ^-1^ cm ^-1^.

### Determination of enzymes activity

To determine enzyme activity, 0.1 g of fresh third leaves were ground in 3 ml of 50 mM Tris-HCl buffer (pH 6.8) at 4˚C. The homogenate was centrifuged at 13000 × g for 20 min at 4˚C. The supernatants were then collected and stored at -70˚C for determination of enzymes activity.

SOD activity was determined using the assay system described by Giannopolitis & Ries (1977). The reaction mixture consisted of 50 mM phosphate buffer pH 7.5, 13 mM methionine, 0.1 mM Na-EDTA, 75µM NBT, 75 µM Riboflavin and 100 µL of enzyme extract in a final volume of 3 ml (all the chemicals were purchased from Sigma-Aldrich). The mixture in glass test tubes was placed 30 cm from 30 W fluorescent lamps. Identical solutions without illumination and enzyme extract were considered as blanks. Since SOD has the ability to inhibit the photochemical reduction of nitroblue tetrazolium (NBT), the amount of inhibition was estimated by reducing the generation of color in the presence of light. One unit of SOD was described as the amount of enzyme that lead to 50% inhibition of NBT reduction. After 16 min, the absorbance at 560 nm was recorded against the blank. SOD activities were calculated as units per milligram of protein.

POX activity was measured based on the method of Abeles & Biles (1991). The activity of POX was estimated by adding 0.01 ml of enzyme extract to 4 ml of 0.2 M acetate buffer (pH 5), 0.4 ml H _2_O _2_ (3%), 0.2 ml 20 mM benzidine. The absorbance was recorded at 530 nm using spectrophotometer and POX activity was expressed as U mg ^-1^ protein.

PPO was assayed according to Raymond *et al*. (1993). The reaction solution contained 2.5 ml of 200 mM sodium phosphate buffer (pH 6.8), 0.2 ml of 20 mM pyrogallol and 0.01 ml enzyme extract. The temperature of the reaction mixture was 40˚C. The changes in absorbance were recorded at 430 nm.

### Statistical analysis

Each experiment was repeated three times. Data were statistically analyzed using analysis of variance one-way (ANOVA) with SPSS software (version 18). Means were compared by Duncan’s test at the 0.05 level of confidence.

## Results

**Figure 1.**
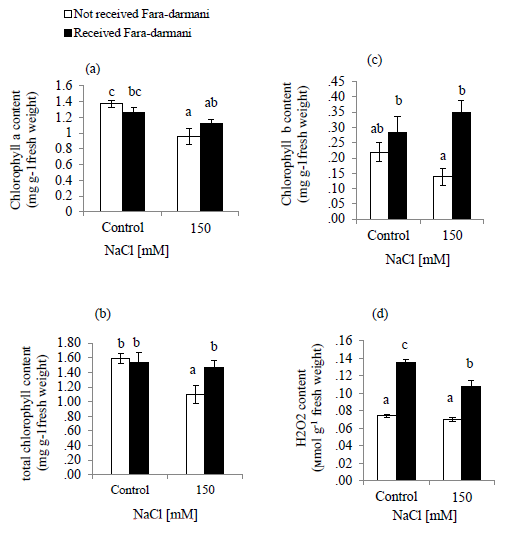
Effects of Fara-darmani Consciousness Field treatment on ( **a**) chlorophyll a, ( **b**) total chlorophyll, ( **c**) chlorophyll b, ( **d**) hydrogen peroxide (H _2_O _2_). Plants were treated with 0 mM NaCl (control) or 150mM NaCl *Vertical bars* indicate mean ± standard error of three replicates. Means followed by the same *lette*r were not significantly different at P<0.05.

Salinity decreased the contents of chlorophyll (Chl) a, Chl b and total Chl ( Figure 1a–c). Under the influence of Fara-darmani CF with 150 mM NaCl, the contents of total Chl, Chl a and Chl b were elevated (34.8%, 17.8% and 169%, respectively) compared to the plants treated with 150mM without Fara-darmani CF.

The effect of NaCl treatment on H _2_O _2_ is shown in ( Figure 1d). Results of the present study showed that H _2_O _2_ content remained unchanged under salinity condition whereas for the Fara-darmani CF treated groups (control and 150mM NaCl) showed significant enhancement 100% and 57.1%, respectively.

**Figure 2.**
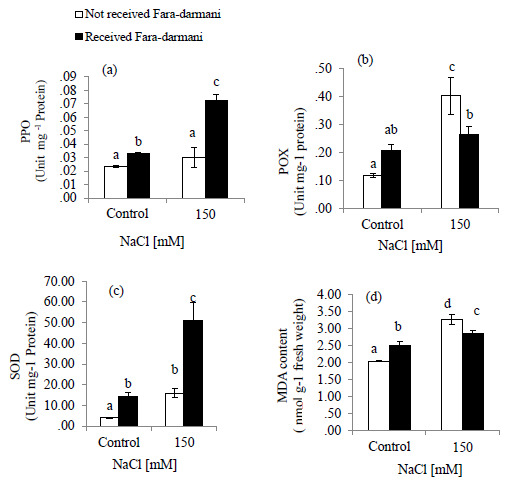
Effects of Fara-darmani Consciousness Field treatment on antioxidant enzyme activities. ( **a**) polyphenol oxidase (PPO), ( **b**) peroxidase (POX), ( **c**) superoxide dismutase (SOD) and ( **d**) malondialdehyde (MDA) content. Plants were treated with 0 mM NaCl (control) or 150mM NaCl *Vertical bars* indicate mean ± standard error of three replicates. Means followed by the same *lette*r were not significantly different at P<0.05.

MDA content was assessed as an oxidative indicator. Salinity stress caused an increase of 59.5% in MDA content as compared to that of control. The Fara-darmani CF treatment to the salt-stressed plant decreased MDA content by about 12.5% ( Figure 2d). 

POX activity was significantly increased by NaCl treatment up to 244 % compared with control while under salinity treatment exposure to Fara-darmani CF decreased the activity of enzyme by 34 % ( Figure 2b).

SOD activity was slightly increased under salinity. However, it was found that with Fara-darmani CF the activity of SOD in salinity condition was about 220 % higher than that in salinity without Fara-darmani CF treatment ( Figure 2c)

Similarly, PPO activity was not significantly higher than non-saline condition (control). However, the PPO activity showed an increase of 168% under salinity in response to Fara-darmani CF compared to the salinity treated without Fara-darmani CF treatment ( Figure 2a).

## Discussion

In this study, chlorophyll a and b, and total chlorophyll contents decreased remarkably under salinity conditions ( Figure 1). This is supported by previous data reported in tomato plants ( Al-aghabary *et al*., 2005) and wheat ( Ashraf *et al*., 2002), where salt stress unfavorably affects chlorophyll content. The decrease in chlorophyll content might be due to the formation of ROS in salinity stress that leads to lipid peroxidation and damages thylakoid membranes ( Mittler, 2002). Our results showed that under salinity treatment, Fara-darmani CF ameliorated the adverse effects of salt stress, probably by improving antioxidant systems, scavenging ROS and increasing the chlorophyll a and b contents ( Figure 1). Similar findings were presented by screening the effect of the Static Magnetic Field (SMF) on pigments in *Lettus* and it has been reported that all photosynthetic pigments, especially chlorophyll a increased under the influence of SMF and this positive result was associated with improvement of antioxidant system ( Latef *et al.*, 2020).

For many years, numerous investigations have been made into salinity stresses and attempt to alleviate detrimental effects of salt stress such as effect of phytohormones, osmoprotectant, antioxidants, polyamines and trace elements ( Kamran *et al.*, 2019). For instance, treatment of 2-week-old rice plants under 150 mM NaCl stress with sodium selenate (Na _2_SeO _4_) improved the total biomass and increased the activity of antioxidant enzyme ( Subramanyam *et al.*, 2019) or exogenous melatonin pretreatment mitigates salt stress in wheat seedlings through regulation on polyamine metabolism ( Ke *et al.*, 2018). In addition, it has been reported that exogenous application of ascorbic acid could alleviate the adverse effects of salt stress on wheat plant ( Athar *et al.*, 2008; Khan *et al.*, 2006). In this research, for the first time we investigate the effects of Fara-darmani CF on wheat plant in order to improve plant tolerance to salinity stress. Although, consciousness field is neither matter nor energy, it is possible to trace its effects with various experimental test. For instance, the changes in cancer cell growth ( Taheri *et al.*, 2020a) and investigation on the electrical activity of the brain during Fara-darmani connection in the Fara-therapist population ( Taheri *et al.*, 2020b) are other observations that have used this method in research.

Various abiotic stresses, including salinity, contribute to formation of ROS ( Navari-Izzo *et al*., 1998). Data of this study showed that under salinity conditions there was an increase in H _2_O _2_ content with Fara-darmani CF treatment, which coincided with an increase in SOD activity (about 220%). Similarly, it has been reported that Static Magnetic Field (SMF), induced H _2_O _2_ content and SOD activity in *Matricaria chamomilla* ( Hassanpour & Niknam 2020). SOD coverts superoxide radicals to H _2_O _2_ and molecular oxygen. It is possible that increasing H _2_O _2_ could therefore be attributed to Fara-darmani-induced enhancement of SOD activity. This function may have a key role in mitigating oxidative stress. SOD is the first enzyme involved in antioxidative processes ( Rubio *et al*., 2002). Increasing the activity of SOD was observed similarly in the leaves of sugar beet ( Bor *et al*., 2003) Lycopersicon ( Koca *et al*., 2006), and in wheat ( Esfandiari *et al*., 2007; Sairam *et al*., 2002) under salt stress. However, under salinity conditions, Fara-darmani CF decreased POX activity, which decomposes the H _2_O _2_ produced by SOD. Wang *et al*. (2014) demonstrated that the activity of POX decreased in four wheat cultivars under high temperature (HT) stress and HT-resistant wheat cultivars had higher SOD activity. These results suggest that H _2_O _2_ may take part in the signaling networks. It has been reported that seed pretreatment with H _2_O _2_ improves salt tolerance of wheat seedlings by alleviation of oxidative damage and expression of stress proteins ( Wahid *et al*., 2007). Additionally, accumulation of H _2_O _2_ is thought to be a signal for induction of pathogenesis-related (PR) genes ( Chen *et al*., 1993). Kuźniak & Urbanek (2000) reported that H _2_O _2_ contributes to signal transduction, gene expression and cellular defense under oxidative stress conditions.

In the present study, Fara-darmani CF also induced PPO activity. PPO may play a key role in scavenging H _2_O _2_ in salt-stressed plant. Agarwal & Pondey (2004) found that in *Cassia angustifolia* PPO activity increased under salinity stress. In wheat, various experiments reported changes in antioxidant enzymes activities to overcome environmental stresses ( Agarwal *et al*., 2005; Barakat, 2011; Heidari, 2009). The mechanism of action of Fara-darmani CF as an inducer of antioxidant enzymes activity is not clear; therefore, future studies are needed to gain additional insights on biological and biochemical effects of this CF on various plants under biotic and abiotic stresses.

MDA content, which is a product of lipid peroxidation, reflects membrane destruction under oxidative stresses ( Hernández & Almensa, 2002). Increase of MDA content under salt stress have been previously observed in different studies ( Hasanuzzaman *et al*., 2014; Saha *et al*., 2010; Torabi & Niknam, 2011; Weisany *et al*., 2012). Mandhania *et al*., 2006, reported that the better NaCl tolerance in a salt-tolerant cultivar of wheat was associated with lower MDA and much higher activities of antioxidant enzymes compared to salt-sensitive cultivar. Fara-darmani as a CF decreased MDA content under salinity stress. It seems that decreased MDA content is correlated with increased activity of antioxidant enzymes under the influence of Fara-darmani CF and a strategy developed by plant to withstand salt stress. 

From these results, it can be concluded that Fara-darmani CF minimizes the negative effects of salt stress in the wheat plant with evidence of increased activity of antioxidant enzymes, increased chlorophyll content and less membrane damage. The main challenge of this study is the fact that Consciousness Field doesn’t possess a quantity and isn’t directly measurable. Therefore, in order to identify its specific effects, we have measured Fara-darmani CF effects indirectly on a plant’s biochemical processes. We suggest that other researchers repeat similar experiment with different plants. It seems that botanical bioassays are suitable for screening the effect of such treatments, and apart from the placebo responses by humans, these assays can be beneficial to save time and resources.

## Data availability

### Underlying data

Harvard Dataverse: Alleviative Effects of Fara-darmani Consciousness Field on Triticum aestivum L. under Salinity Stress, https://doi.org/10.7910/DVN/XNMRMV ( Torabi, 2020).

This project contains the following underlying data:

- Raw data of chlorophyll a, chlorophyll b, hydrogen peroxide, MDA, POX, PPO, SOD, and total chlorophyll content in control and salinity conditions with and without receiving Fara-darmani consciousness field (separate .tab files).
- Charts of chlorophyll a, chlorophyll b, hydrogen peroxide, MDA, POX, PPO, SOD, and total chlorophyll content in control and salinity condition with and without receiving Fara-darmani consciousness field (separate .docx files).

Data are available under the terms of the Creative Commons Zero "No rights reserved" data waiver (CC0 1.0 Public domain dedication).
