# Supplementary material for: Alleviative effects of Fara-darmani Consciousness Field on Triticum aestivum L. under salinity stress
Source: F1000Res. 2020 Dec 2;9:1089. [Version 1] doi: 10.12688/f1000research.25247.2 (PMC10300500; doi:10.12688/f1000research.25247.2)
Supplement: Peer review history

# Peer review: Reviewer response for version 2

**DOI:** 10.5256/f1000research.30995.r75682

**Published:** 2021-01-14

**Authors:** Halimeh Hassanpour

**Affiliations:** 1 Aerospace Research Institute, Ministry of Science Research and Technology, Tehran, Iran

All comments were done in the text, and the art is confirmed for indexing in the journal.

Is the work clearly and accurately presented and does it cite the current literature?

Partly

If applicable, is the statistical analysis and its interpretation appropriate?

Yes

Are all the source data underlying the results available to ensure full reproducibility?

Yes

Is the study design appropriate and is the work technically sound?

No

Are the conclusions drawn adequately supported by the results?

Partly

Are sufficient details of methods and analysis provided to allow replication by others?

No

Reviewer Expertise:

NA

I confirm that I have read this submission and believe that I have an appropriate level of expertise to confirm that it is of an acceptable scientific standard.

# Peer review: Reviewer response for version 1

**DOI:** 10.5256/f1000research.27861.r73503

**Published:** 2020-11-19

**Authors:** Halimeh Hassanpour

**Affiliations:** 1 Aerospace Research Institute, Ministry of Science Research and Technology, Tehran, Iran

This research investigated effect of Fara-darmani Consciousness Field on alleviation of salt stress. It has some interests for the readers and me. Fara-darmani Consciousness Field increased antioxidant systems in plants against stress. But There are some questions about this art, and need to answer by authors:

 Why did the authors select just 0 and 150 mM Nacl for this research?

 The intensity or wavelength are not clear, and isn’t the device regulates for intensity or wave?

 How Fara-darmani Consciousness Field affect the cell, how is perceived by the cell, what is the effect on organisms? The discussion needs to improve.

 If there isn’t any research about Fara-darmani Consciousness Field, the author can use some article for the discussion:

  

- Impact of the Static Magnetic Field on Growth, Pigments, Osmolytes, Nitric Oxide, Hydrogen Sulfide Phenylalanine Ammonia-Lyase Activity, Antioxidant Defense System, and Yield in Lettuce. ^1^
- Establishment and assessment of cell suspension cultures of *Matricaria* *chamomilla* as a possible source of apigenin under static magnetic field **.** ^2^

Is the work clearly and accurately presented and does it cite the current literature?

Partly

If applicable, is the statistical analysis and its interpretation appropriate?

Yes

Are all the source data underlying the results available to ensure full reproducibility?

Yes

Is the study design appropriate and is the work technically sound?

No

Are the conclusions drawn adequately supported by the results?

Partly

Are sufficient details of methods and analysis provided to allow replication by others?

No

Reviewer Expertise:

NA

I confirm that I have read this submission and believe that I have an appropriate level of expertise to confirm that it is of an acceptable scientific standard, however I have significant reservations, as outlined above.

## References

[1] : Establishment and assessment of cell suspension cultures of Matricaria chamomilla as a possible source of apigenin under static magnetic field. *Plant Cell, Tissue and Organ Culture (PCTOC)* .2020;142(3) : 10.1007/s11240-020-01885-4 583-593 10.1007/s11240-020-01885-4

[2] : Impact of the Static Magnetic Field on Growth, Pigments, Osmolytes, Nitric Oxide, Hydrogen Sulfide, Phenylalanine Ammonia-Lyase Activity, Antioxidant Defense System, and Yield in Lettuce. *Biology (Basel)* .2020;9(7) : 10.3390/biology9070172 10.3390/biology9070172 32709036PMC7408432

# Response

**Published:** 2020-11-22

**Authors:** Sara Torabi

**Affiliations:** University of Tehran, Iran

**Dear Dr. Hassanpour**

 Thank you very much for your kind words about our paper. In the following sections, you will find our responses to each point and suggestions. we have carefully reviewed the comments and have revised the manuscript accordingly.

**Why did the authors select just 0 and 150 mM Nacl for this research?**

 In the past, various investigations have been made into salinity stresses and attempt to minimize the losses of plant productivity, such as the effects of Coumarin (Saleh and Madany, 2015), Ascorbic acid (Athar et al, 2008, Azzedin et al, 2011, Khan et al, 2006), Oligochitosan (Ma, et al, 2012), Melatonin (Ke, et al, 2018), etc. on the wheat plant. Therefore, based on previous investigations, we screened ameliorative effects of Fara-darmani CF with two similar concentrations of NaCl including 0 and 150 mM. We suggest that the effects of the Fara-darmani CF be investigated with different NaCl concentrations and with different types of plants.

 Ahmed Mahmoud Saleh, M.M.Y. Madany. Coumarin pretreatment alleviates salinity stress in wheat seedlings, Plant Physiology and Biochemistry, Volume 88, 2015, Pages 27-35, ISSN 0981-9428, https://doi.org/10.1016/j.plaphy.2015.01.005.

 Habib-ur-Rehman Athar, Ameer Khan, Muhammad Ashraf, Exogenously applied ascorbic acid alleviates salt-induced oxidative stress in wheat, Environmental and Experimental Botany, Volume 63, Issues 1–3, 2008, Pages 224-231, ISSN 0098-8472, https://doi.org/10.1016/j.envexpbot.2007.10.018

 Azzedine, F, Gherroucha, H, Baka, M. Improvement of salt tolerance in Durum Wheat by Ascorbic acid application. Journal of Stress Physiology & Biochemistry, Vol. 7, 2011, No. 1, pp. 27-37 ISSN 1997-0838

 Khan A, Ahmad MSA, Athar HUR, Ashraf M. Interactive effect of foliarly applied ascorbic acid and salt stress on wheat (Triticum aestivum L.) at the seedling stage. Pakistan Journal of Botany. 2006 Dec;38(5):1407-1414.

 Ma, L., Li, Y., Yu, C. *et al.* Alleviation of exogenous oligochitosan on wheat seedlings growth under salt stress. *Protoplasma* **249, **393–399 (2012). https://doi.org/10.1007/s00709-011-0290-5

 Ke Qingbo, Ye Jun, Wang Bomei, Ren Jianhong, Yin Lina, Deng Xiping, Wang Shiwen. Melatonin Mitigates Salt Stress in Wheat Seedlings by Modulating Polyamine Metabolism, Frontiers in Plant Science, VOLUME 9, 2018, PAGES 914. ISSN 1664-462X. DOI: 10.3389/fpls.2018.00914.

**The intensity or wavelength are not clear, and isn’t the device regulates for intensity or wave?**

 As it was mentioned in the introduction section (paragraph 4, lines 5), according to Taheri, consciousness is one of the three existing elements of the universe apart from matter and energy. By defining consciousness as neither matter nor energy we cannot associate a quantity to it. Therefore, we cannot register it by any device and must be mediated by human mind.

**How Fara-darmani Consciousness Field affect the cell, how is perceived by the cell, what is the effect on organisms? The discussion needs to improve**.

 As stated in the last paragraph, the main challenge of this study is the fact that Consciousness Field doesn’t possess a quantity and isn’t directly measurable. Therefore, in order to identify its specific effects, we have measured Fara-darmani CF effects indirectly on a plant’s biochemical processes. At this point, more biochemical and histological studies can collaborate to clarify this phenomenon. In addition, more explanation was added in the discussion section (paragraph 2).

**If there isn’t any research about Fara-darmani Consciousness Field, the author can use some article for the discussion:**

 Thank you for this suggestion. We have put a significant effort in improving the discussion section and these two references were added to the paragraph 1 and 2. In addition, we improved the discussion with more references.
